# Smart Parasitic Nematodes Use Multifaceted Strategies to Parasitize Plants

**DOI:** 10.3389/fpls.2017.01699

**Published:** 2017-10-04

**Authors:** Muhammad A. Ali, Farrukh Azeem, Hongjie Li, Holger Bohlmann

**Affiliations:** ^1^Department of Plant Pathology, University of Agriculture Faisalabad, Faisalabad, Pakistan; ^2^Centre of Agricultural Biochemistry and Biotechnology, University of Agriculture Faisalabad, Faisalabad, Pakistan; ^3^Department of Bioinformatics and Biotechnology, Government College University, Faisalabad, Pakistan; ^4^National Key Facility for Crop Gene Resources and Genetic Improvement, Institute of Crop Science, Chinese Academy of Agricultural Sciences, Beijing, China; ^5^Division of Plant Protection, Department of Crop Sciences, University of Natural Resources and Life Sciences, Vienna, Vienna, Austria

**Keywords:** PPNs, effector proteins, compatible interaction, molecular parasitism, cyst and root-knot nematodes

## Abstract

Nematodes are omnipresent in nature including many species which are parasitic to plants and cause enormous economic losses in various crops. During the process of parasitism, sedentary phytonematodes use their stylet to secrete effector proteins into the plant cells to induce the development of specialized feeding structures. These effectors are used by the nematodes to develop compatible interactions with plants, partly by mimicking the expression of host genes. Intensive research is going on to investigate the molecular function of these effector proteins in the plants. In this review, we have summarized which physiological and molecular changes occur when endoparasitic nematodes invade the plant roots and how they develop a successful interaction with plants using the effector proteins. We have also mentioned the host genes which are induced by the nematodes for a compatible interaction. Additionally, we discuss how nematodes modulate the reactive oxygen species (ROS) and RNA silencing pathways in addition to post-translational modifications in their own favor for successful parasitism in plants.

## Introduction

Phytoparasitic nematodes are a serious menace to world's food security. They are biotrophic obligate parasites, whether migratory or sedentary in parasitic behavior (Ali et al., 2017). In both cases they consume the cell sap and plant nutrients during the course of parasitism. Around 4,300 species have been reported as plant parasitic nematodes (PPNs), which account for 7% of the phylum Nematoda (Decraemer and Hunt, 2006). The sedentary phytoparasitic nematodes develop compatible interactions with a wide range of crop plants including wheat (*Triticum aestivum* L.), potato (*Solanum tuberosum* L.), tomato (*S. lycopersicum* L.), soybean [*Glycine max* (L.) Merr.] and sugar beet, (*Beta vulgaris* L.). The worldwide crop losses incurred by parasitic nematodes are estimated to be over 157 million dollars annually (Abad et al., 2008). Cyst and root-knot nematodes belonging to the family *Heteroderidae* are the most important categories of phytoparasitic nematodes which parasitize a large number of plant species. The root-knot nematode, *Meloidogyne incognita* (Kofoid and White) Chit. alone infects more than 3,000 plant species including several crop plants (Abad et al., 2008), where nematodes of the genus *Meloidogyne* induce the development of root galls or knot cells (Jones and Payne, 1978). On the other hand, cyst nematodes from genera *Heterodera* and *Globodera* establish syncytia in plant roots (Jones, 1981). We have reviewed in detail the physiological and molecular events involved in the development of these nematode feeding sites (NFSs; Ali M. A. et al., 2015).

Since the sequencing of the genome of *M. incognita* (Abad et al., 2008) our understanding has significantly been increased regarding nematode genes and proteins that are involved in successful plant-nematode interactions. Among enormous researches on plant-nematode interactions, a lot of information is available about the genes and proteins that determine nematode virulence (reviewed by Bellafiore and Briggs, 2010). The PPNs have developed several strategies to parasitize plants by using their secretions. These secretions include cell wall degrading enzymes (CWDEs), effectors and proteins involved in mimicry of host proteins for success of nematode establishment on plants (Huang et al., 2003; Bellafiore et al., 2008; Caillaud et al., 2008). The functional roles of various nematode effectors in compatible and incompatible interactions have been reviewed before (Gheysen and Vanmontagu, 1995; Vanholme et al., 2004; Gheysen and Mitchum, 2011; Haegeman et al., 2012; Jaouannet and Rosso, 2013; Kyndt et al., 2013; Mitchum et al., 2013).

PPNs are able to reprogram the expression of plant genes in their own favor using their secretions (Ali M. A. et al., 2015). They are able to induce the expression of genes which are important for their establishment on the plant roots. Nematode induced feeding cells are metabolically hyperactive sites which are the only source of nutrients for the nematodes (Siddique et al., 2009, 2012; Szakasits et al., 2009; Hofmann et al., 2010). On the other hand, nematodes are also able to suppress the expression of defense related genes to avoid resistance responses of their hosts (Gheysen and Fenoll, 2002; Ali M. A. et al., 2015). This review is an update from the most recent literature on how the nematode secretome is involved in establishing successful parasitism in plants and how they manipulate various defense related pathways in plants to avoid resistance responses.

## The nematode secretome contains a variety of effectors

**Figure 1:**
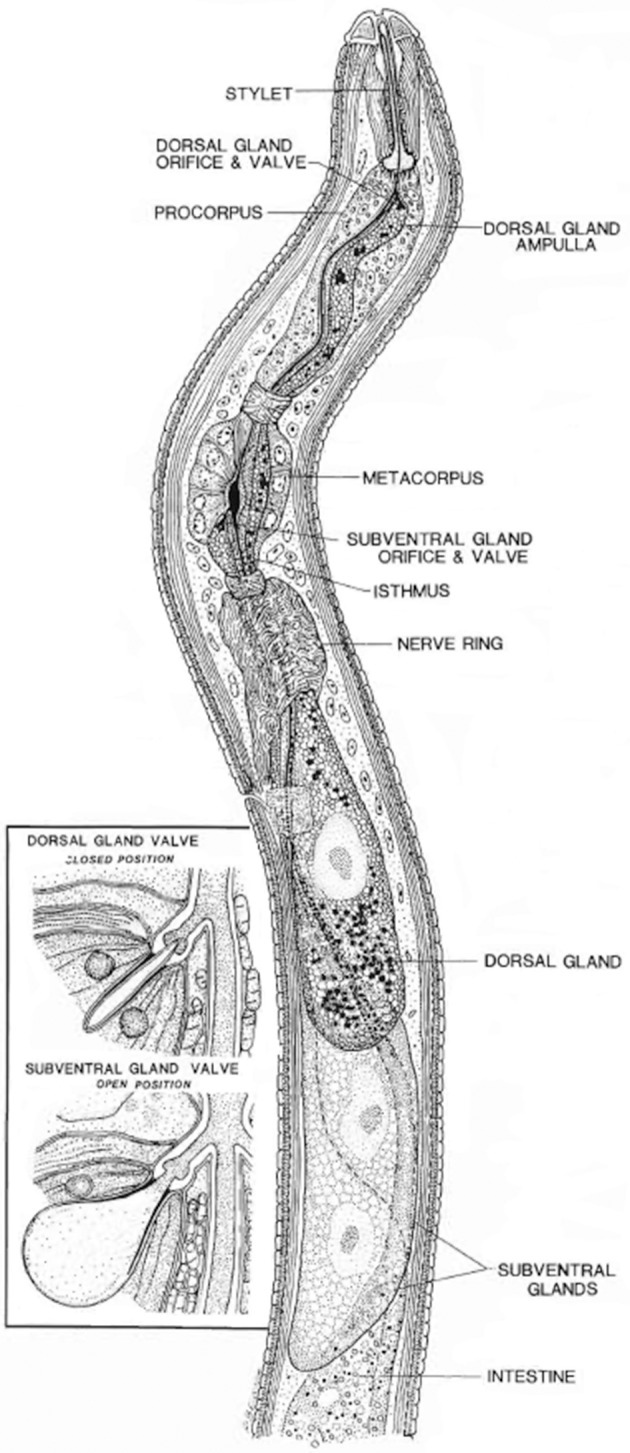
Longitudinal view through the anterior region of a juvenile soybean cyst nematode. The insert shows a closed valve or end apparatus within a dorsal gland ampulla and the open valve or end apparatus within one of a pair of subventral gland ampullae (Endo, 1984). Reproduced with permission of the Helminthological Society of Washington.

All PPNs have a unique tool, a needle like structure called stylet, to puncture cell walls and to introduce their secretions into the host plant cells to control the complex parasitism process (Davis et al., 2000). A small proportion of these secretions also come from nematode amphids and cuticle. The proteins secreted by nematodes through the stylet are primarily important for early infection and nematode establishment on the plants; however, these proteins differ significantly in various nematode developmental and parasitic stages (Hussey, 1989; Davis et al., 2000). These secretions are collectively called “secretome” (secreted proteins) of nematodes that results in successful parasitism in plants (Hussey et al., 2002). The effector proteins which are directly or indirectly involved in parasitism are collectively known as “parasitome.” Moreover, for parasitic success, the nematodes have evolved in their morphology and physiology, i.e., significant modifications in esophagus at different growth stages (Hussey, 1989; Figure 1).

The secretory gland cells of the nematodes are the main source of effector proteins (Figure 1). Both synthesis and secretion of effector proteins are developmentally regulated in the esophageal glands at different parasitic stages of the nematode (Wyss and Zunke, 1986; Davis et al., 2008). It is assumed that the secretions produced by nematodes in their esophageal gland cells induce the differentiation of root cells in the vicinity of vasculature into complex feeding sites (reviewed by Ali M. A. et al., 2015).

The secretomes of sedentary endoparasitic nematodes, especially members of the genera *Meloidogyne, Heterodera*, and *Globodera*, are particularly interesting because they induce drastic modifications of gene expression in the parasitized plant cells leading to complex morphological, biochemical, and metabolic changes which turns the parasitized root cells into unique NFSs (Gheysen and Fenoll, 2002). The secretome carries typically a huge volume of highly concentrated proteins; however, the number and amount of various secretory proteins in the matrix may differ with different gland cell types at various developmental stages (Burgess and Kelly, 1987; Vanholme et al., 2004). The sedentary nematodes like root–knot nematodes (RKNs) and cyst nematodes (CNs) deposit their secretions either outside the plasma membrane or inject them straight into the cytoplasm of the recipient cell through their stylet. In both cases, some proteins may mimic the signaling cascade leading to the modification of gene expression of the host cells (Davis and Mitchum, 2005; Mitchum et al., 2013).

Gao et al. (2003) worked out the first comprehensive parasitome profile for the soybean cyst nematode, *H. glycines* Ichinohe. They reported 51 gland-expressed candidate parasitism genes which were previously unidentified. Individual proteins from the parasitome exhibited distinct gland cell expression patterns throughout different parasitic stages (Gao et al., 2003). Furthermore, in the secretome of *M. incognita* 486 secreted proteins were identified. Most of these proteins are homologous to plant proteins, which may be stimulated or induced, and some are effectors which result in the modulation of the plant cell cycle, while others could reprogram the expression of host genes to favor parasitism (Bellafiore et al., 2008). Peng et al. (2013) have explored the host parasitism of the migratory potato rot nematode *Ditylenchus destructor* Thome by expressed sequence tags (EST) analysis. They reported that 22 ESTs were found similar to effectors reported from other nematodes and most of these effectors were involved in host cell wall degradation and/or modification i.e., 1,4-beta-glucanse, 1,3-beta-glucanase, pectate lyase, chitinases, and expansin, however, some of them might be involved in host defense suppression i.e., annexin, calreticulin, and venom allergen-like protein (Peng et al., 2013).

Most parasitism genes encode proteins that contain a signal peptide which targets the protein to the endoplasmic reticulum and through the secretory pathway. Interestingly, some proteins have no signal peptide; for instance, a glutathione-S-transferase (GST) gene which is only expressed in the subventral glands of J3 and encodes a protein that lacked a signal peptide (Dubreuil et al., 2007). This suggests that the proteins lacking signal peptide could be secreted from parasitic nematodes via different secretory pathways.

The proteins of the secretome include the following categories of effectors:

1. Cell wall degrading enzymes and cell wall loosening proteins which are important for movement through the plant and help piercing the cell walls.
2. Effectors which induce the development of NFSs within the plant and modify the metabolism of these NFSs to establish a compatible interaction.
3. Suppressors that are important to suppress plant resistance responses.

## Proteins for cell wall degradation and modification

The plant cell wall is the first obstacle faced by PPNs during the course of penetration, infection, and movement into the plant cells. PPNs use their stylet for piercing plant cell walls followed by the secretion of cell wall degrading and modifying enzymes. The structural complication of cell walls is reflected by the variety of CWDEs secreted by PPNs that are able to degrade or change the composition of different structural polysaccharides like cellulose, xylans, hemicellulose, and pectin (Bohlmann and Sobczak, 2014; Wieczorek, 2015). PPNs are different from free living nematodes due to the secretion of CWDEs, which are involved in localized cell wall degradation and modification during the course of infection. These enzymes are very important for movement of nematodes into the plant tissues. Various enzymes are involved in degradation of different carbohydrate polymers which are constituents of the plant cell walls. However, nematodes also use expansins for softening of cell walls for easy movement through the plant tissues. The number of different cell wall degrading and modifying enzymes identified in the genomes of various PPNs is given in Table 1. The most common PPN parasitism genes, which have been extensively studied, code for cellulases or endo-1,4-β-glucanases. The first cellulase from a nematode was reported at the end of last century (Smant et al., 1998), and after that several studies have shown their occurrence in the secretome of various PPNs (Yan et al., 1998; de Boer et al., 1999; Goellner et al., 2000, 2001; De Meutter et al., 2001; Gao et al., 2002, 2004; Kikuchi et al., 2004; Abad et al., 2008; Haegeman et al., 2008, 2010; Kyndt et al., 2008; Rehman et al., 2009a). The function of these enzymes is to degrade and soften the plant cell walls to facilitate the nematode invasion, migration through the plant tissues and ultimately to establish NFSs (Ali M. A. et al., 2015). Cellulases are comprised of a signal peptide and a catalytic domain; however, in some cases, an additional carbohydrate-binding module (CBM) is also present. The majority of the known endoglucanases belong to the glycosyl hydrolase family 5 (GHF5), while some evolutionary divergent species possess GHF45 [*Bursaphelenchus xylophilus* (Steiner and Buhrer) Nickle; Kikuchi et al., 2004] and GHF12 endoglucanases [*Xiphinema index* Thome and Allen (1950); Jones et al., 2005]. Because the proteins coded by endoglucanase genes are very similar to bacterial endoglucanases, it was suggested that these are derived from bacteria through horizontal gene transfer (Jones et al., 2005). Genes coding for another class of enzymes called carbohydrate active enzymes (CAZy) have also been identified in nematode species, i.e., *Pratylenchus penetrans* (Cobb, 1917) Filip. and Stek. 1941, which have probably been horizontally transferred from bacteria (Haegeman et al., 2011). These enzymes are also helpful for the nematodes to develop compatible interactions with the plants.

**Table 1:** Comparison of predicted cell wall degrading enzymes from the genomes of different nematode species.

**Nematode species**	**Size (Mb)**	**Gene models (#genes)**	**Cellulases**	**Xylanases**	**Arabinanases**	**Pectate lyases**	**Expasins**	**Total**	**References**
*Bursaphelenchus xylophilus*	75	18,074	11	0	0	15	8	34	Kikuchi et al., 2011
*Meloidogyneincognita*	86	19,212	21	6	2	32	20	81	Abad et al., 2008
*M. hapla*	53	13,072	6	1	2	24	6	39	Opperman et al., 2008
*Caenorhabditis elegans*	100	20,431	0	0	0	0	0	0	Consortium, 1998
*Pristionchus pacificus*	173	24,216	7	0	0	0	0	7	Dieterich et al., 2008
*Bursaphelenchus mucronatus*	96	21,252	0	0	0	0	0	0	Pereira et al., 2013
*Globodera pallida*	124.7	16,419	16	6	1	7	9	39	Cotton et al., 2014
*Pratylenchus coffeae*	19.67	6,712	1	2	2	4	0	9	Burke et al., 2015
*G. rostochiensis*	95.9	14,309	11	0	0	4	3	18	Eves-Van Den Akker et al., 2016
*Rotylenchulus reniformis*	37.45	10,452	11	1	0	2	3	17	Nyaku et al., 2014

The expansins are mainly involved in loosening of plant cell walls through a non-enzymatic mechanism that prompts slippage of cellulose microfibrils in the plant cell wall (Sampedro and Cosgrove, 2005). The first functional expansin (Gr-EXPB1) was reported in the golden potato cyst nematode *Globodera rostochiensis* (Wollenweber) Behrens (Qin et al., 2004; Kudla et al., 2005). They showed that Gr-EXPB1 can disrupt covalent bonds in plant cell walls along with its accompanying ability to loosen non-covalent bonds. Gr-EXPB1 shows similarity with both bacterial and plant expansins. Moreover, it shows highest sequence similarity to two hypothetical proteins from the aerial mycelium-forming soil-inhabiting Actinobacteria and *Streptomyces lavendulae*. However, the 2nd domain of Gr-EXPB1 (residues 150–271) showed substantial similarity to a β-expansin-like protein (PPAL) from *Nicotiana tabacum* L. and a putative β-expansin from *Arabidopsis thaliana* L. (Qin et al., 2004; Kudla et al., 2005). Another expansin-like protein MAP-1 was identified in *M. arenaria, M. incognita*, and *M. javanica*. Polyclonal antibodies were produced against MAP-1 peptide which strongly labeled J2 amphidial secretions in immunofluorescence microscopy assays, suggesting that MAP-1 might have a role in the early steps of plant-nematode interactions (Semblat et al., 2001). Recently, a large MAP-1 gene family has been identified in the genus *Meloidogyne*. The members of this family code for expansin-like proteins which are secreted into plant tissues during parasitism, also thought to function as effectors to stimulate successful root infection (Tomalova et al., 2012). The phylogenetic studies and the distribution of *map-1* genes in RKNs further indicated the presence of these genes in species that reproduce by mitotic parthenogenesis (Tomalova et al., 2012). Haegeman et al. (2009) identified a putative expansin like gene (*Da-exp1*) in the migratory nematode *Ditylenchus africanus*. Similarly, the transcripts encoding expansin-like proteins were discovered in the transcriptome of *P. penetrans* which showed high similarity with expansin-like genes from *H. avenae* and *H. glycines* (Vieira et al., 2015). However, *P. coffeae*, which harbors the smallest genome, lacks expansins (Burke et al., 2015). In addition to cell wall modification and softening, an expansin-like protein from *G. rostochiensis* (*GrEXPB2*) has shown suppression of cell death induced by NB-LRR proteins in different *Nicotiana* spp. (Ali S. et al., 2015). Moreover, this protein was found to be involved in the R genes mediated suppression of immune response in plants, suggesting its role as a negative modulator of nematode parasitism (Ali S. et al., 2015). More recently, an expansin like gene from *H. avenae* (*HaEXPB2*) has been reported to be involved in compatible interaction as silencing of this gene resulted in reduced *H. avenae* infectivity (Liu et al., 2016).

Pectate lyases or pectinases catalyze the cleavage of internal α-1,4-linkages of pectate by β-elimination and have a potential role in pectin degradation (Barras et al., 1994). These are classified as polysaccharide lyases and are the members of 5 from 15 families (1, 2, 3, 9, and 10) of this type of enzymes (Henrissat, 1991; Henrissat and Davies, 1997). Pectinases are also the part of the nematode parasitomes secreted from the stylet and attack the host cell wall (Davis et al., 2004). These CWDEs are not present in free living, non phytoparasitic nematodes, or any other animals. These are very similar to bacterial pectate lyase proteins, so it is thought that nematodes might have acquired them by horizontal gene transfer (HGT) from bacteria (Jones et al., 2005). Two pectate lyase genes (*Bx-pel-1* and *Bx-pel-2*) were identified and characterized from the pine wood nematode, *B. xylophilus* (Kikuchi et al., 2006). These are expressed solely in the esophageal gland cells of the nematode, showing their secretion into plant tissues to support feeding and migration in the tree. Geric Stare et al. (2011) analyzed the polymorphisms of pectate lyase2 (*pel-2*) gene in the genus *Globodera* and obtained 78 different *pel-2* sequences from populations of *G. rostochiensis, G. pallida, G. Mexicana*, and *G. tabacum*. It suggests that pectate lyases are widely distributed in this genus and might play an important role in compatible plant-nematode interactions. Similarly, four transcripts were found for polygalacturonases (GH28) in the transcriptome of *P. penetrans* infecting the soybean roots (Vieira et al., 2015). Widespread genome and RNAseq sequencing is now uncovering a large number of genes coding for CWDEs. However, sequence similarity is not always a reliable indicator of enzymatic function. The actual function has to be determined with wet lab experiments. Nematode secreted CWDEs along with their subcellular localization and putative function are shown in Table 2. For in depth information please consult the recently developed Nematode CWDE database (Rai et al., 2015).

**Table 2:** Cell wall degrading and modifying enzymes secreted by nematodes into the plant roots.

**Gene name**	**Nematode species**	**Putative function**	**Cellular localization**	**References**
*Gr-eng-1, Gr-eng-2 and Hg-eng-1, Hg-eng-2*	*Globodera rostochiensis* and *Heterodera glycines*	Cellulases, β-1,4-endoglucanases (cellulose digestion, cell wall degradation, modification, and intracellular migration)	Subventral gland cells of second-stage juveniles (J2s)	Smant et al., 1998
*Hg-eng-2 and Hg-eng-3*	*H. glycines*	β-1,4-endoglucanases (cell wall degradation, modification, and intracellular migration)	Subventral esophageal glands of J2s	Yan et al., 1998
*Hg-eng-2-like*	*H. glycines*	Endoglucanases (cell wall degradation, modification, and intracellular migration)	Subventral esophageal glands of J2s	Yan et al., 2001
*Hg-eng-4*	*H. glycines*	Endoglucanases	Subventral gland cells of pre-parasitic and migratory parasitic second-stage juvenile	Gao et al., 2002
*Gt-eng-1* and *Gt-eng-2*	*G. tabacum*	Endoglucanases	Subventral esophageal glands of J2s	Goellner et al., 2000
*Da-eng-1*	*Ditylenchus africanus*	Endoglucanases	Subventral esophageal glands	Kyndt et al., 2008
*Pc-eng1*	*Pratylenchus coffeae*			
*Rr-eng-1*	*Rotylenchulus reniformis*	Endoglucanases	Expressed in the J2 and adult vermiform life-stages	Wubben et al., 2010
*Da-engdel1, 2, 3*, and *4*	*D. africanus*	Endoglucanases	Dorsal or subventral gland cells	Haegeman et al., 2010
*Bx-eng-1, 2* and *3*	*Bursaphelenchus xylophilus*	Endoglucanases	Esophageal glands	Kikuchi et al., 2004
*Da-exp1*	*D. africanus*	Expansins loosening and re-arrangement of plant cell wall polysaccharides	Dorsal or subventral gland cells	Haegeman et al., 2010
*Bx-eng-1, 2* and *3*	*B. xylophilus*	Endoglucanases (weakening of the mechanical strength of the cell walls)	Esophageal gland cells	Shibuya and Kikuchi, 2008
*gr-ams-1*	*G. rostochiensis*	β-1,4, endoglucanases essential for sense organ function	Sheath cells of the amphids	Chen et al., 2005
*Mi-eng-1*	*M. incognita*	β-1,4, endoglucanases (nematode specific plant tissue alterations)	Esophageal gland cells of J2s	Rosso et al., 1999; Béra-Maillet et al., 2000
*Hg-eng-1* and *2*	*H. glycines*	β-1,4, endoglucanases (soften the walls of root cells during penetration and intracellular migration)	Localized along the juvenile's migratory path	de Boer et al., 1999; Wang et al., 1999
*ghf12*	(*Xiphinema index*)	Endoglucanases from GHF family		Jones et al., 2005
*Gr-exp1, Gr-expB1*	*G. rostochiensis*	Expasin (loosens covalent bonds in plant cell walls)	Subventral gland of J2s	Qin et al., 2004; Kudla et al., 2005
*Bx-lam-16A*	*B. xylophilus*	β-1,3-glucanase/cellulase	Oesophageal gland cells	Kikuchi et al., 2005
*Bx-pel-1*and*Bx-pel-2*	*B. xylophilus*	Pectatelyases (help feeding and migration)	Subventral glands of J2s	Kikuchi et al., 2004, 2006
*Mi-pel-1* and *Mi-pel-2*	*M. incognita*	Pectatelyases (facilitate the penetration and intercellular migration by cell-wall-degradation)	Esophageal gland cells of J2s	Huang et al., 2003
*Hg-pel-1*	*H. glycines*	Pectatelyases (cell-wall-degrading and migration)	Subventral glands of J2s	de Boer et al., 2002
*Gr-pel-1*	*G. rostochiensis*	Pectatelyases (cell-wall-degrading and migration)	Subventral oesophageal glands of J2s	Popeijus et al., 2000
*Gr-pel-2*	*G. rostochiensis*	Pectatelyases (cell-wall-degrading and migration)	Subventral oesophageal glands of J2s	Kudla et al., 2007
*Mi-pel3*	*M. incognita*	Cell wall modifications	J2s subventral oesophageal glands along their cytoplasmic extensions, and in the ampullae	Vieira et al., 2011
Polygalacturonases (GH28)	*P. penetrans*	Unknown	2nd stage juveniles	Vieira et al., 2015
*HaEXPB2*	*H. avenae*	Involvement in successful compatible interaction	J2s subventral oesophageal glands	Liu et al., 2016

## Nematode effectors mimic host genes for parasitic success

As mentioned in previous sections, sedentary nematodes induce drastic changes in the root cells to create the so-called NFSs known as syncytia or giant cells. These changes are brought about by the secretions of nematodes delivered into the host root cells. In addition to secreting a mixture of CWDEs and related proteins, the most sophisticated parasitic approach of these sedentary parasites involves manipulating the gene expression of the host cell through a set of effector proteins (Davis et al., 2008; Gheysen and Mitchum, 2011). The effector proteins involved in compatible interactions from different PPNs, their sub-cellular localization and putative functions are given in Table 3.

**Table 3:** Nematode effector involved in compatible and incompatible plant-nematode interactions.

*Bx-crt-1*	*Bursaphelenchus xylophilus*	Calreticulin calcium binding protein, cell-to-cell trafficking and differentiation of NF cells.	Esophageal gland of J2s	Li et al., 2011
*Rs-CRT*	*Radopholus similis*	Essential for the reproduction and pathogenicity	Oesophageal glands and gonads of females and males, the intestines of different juveniles and eggs	Li et al., 2015
*Bx-vap-1*	*B. xylophilus*	Venom allergen-like protein (migration activity of nematodes)	Putative esophageal glands of J2s	Kang et al., 2012
*Mi-vap-2*	*Meloidogyne incognita*	Venom allergen-like protein	Esophageal Gland of J2s	Wang et al., 2007
*Gp-cm-1*	*Globodera pallida*	Chorismate mutases accelerates the conversion of chorismate to prephenate	Subventral gland cells of J2s	Jones et al., 2003
*Mj-cm-1*	*M. javanica*	Accelerates the conversion of chorismate to prephenate	Metacorpus and esophageal gland cells of J2s	Lambert et al., 1999; Doyle and Lambert, 2003
*Hs-UBI1*	*Heterodera schachtii*	Ubiquitin extension protein (regulatory role in NFS formation)	Dorsal pharyngeal gland of J2s	Tytgat et al., 2004
*Hg-chi-1*	*H. glycines*	Chitinase, accumulates specifically in later parasitic stages of *H. glycines* (functional roles in the nematode life cycle)	Subventral oesophageal gland cells	Gao et al., 2002
*Mi-crt*	*M. incognita*	Calreticulin, calcium binding protein (cell-to-cell trafficking and pressure support), key effector in plant defense suppression	Subventral oesophageal gland region	Jaubert et al., 2002, 2005; Jaouannet et al., 2012
*Mi-msp-1*	*M. incognita, M. arenaria*, and *M. javanica*	Venom allergen AG5-like gene with unknown function	Parasitic J2s	Ding et al., 2000
*Gr-TpX*	*G. rostochiensis*	Peroxiredoxin-(defense against very different host responses like ROS)	Nematode surface and material shed from the surface	Robertson et al., 2000
*CLAVATA3 (CLV3)/ESR (CLE)-like effector proteins*	*H. schachtii*	Act as mimics of plant CLE peptides and are required for successful nematode infection	Dorsal gland extension and in the base of the nematode stylet	Replogle et al., 2011
*19C07*	*H. schachtii*	Effector protein 19C07 interacts with the Arabidopsis auxin influx transporter LAX3 to control feeding site development	Dorsal gland of all parasitic stages	Lee et al., 2011
*Hs4F01*	*H. schachtii*	An annexin-like effector may mimic and interact plant annexin function during the parasitic interaction	Dorsal oesophageal gland secretory cell of a third-stage juvenile (J3)	Patel et al., 2010
*SPRYSEC*	*G. rostochiensis*	A secreted spry domain-containing protein (SPRYSEC) binds to the immune receptor SW5-F (CC-NB-LRR) to downregulate its activity	Dorsal esophageal gland of J2s	Rehman et al., 2009b
*Gp-Rbp-1*	*G. pallida*	SPRYSEC protein RBP-1 elicits Gpa2-and RanGAP2-dependent plant cell death	Dorsal esophageal gland	Sacco et al., 2009
*HgCLE1*	*H. glycines*	Protein trafficking and host-specific recognition	Ampulla and dorsal gland cells	Wang J. et al., 2010
*HsCLE1 and HsCLE2*	*H. schachtii*	Mimick target peptides, AtCLEs1–7 to promote parasitism	Dorsal gland cells of J2s	Wang et al., 2011
*Gr-CLE-1 Gr-CLE-4*	*G. rostochiensis*	CLE signaling to facilitate parasitism	Dorsal gland cells of all parasitic stages	Lu et al., 2009
*Rr-cle-1, Rr-cle-2, and Rr-cle-3*	*Rotylenchulus reniformis*	Governing host range and facilitating syncytium formation	Dorsal esophageal gland cell	Wubben et al., 2015
*Mj-nulg1a*	*M. javanica*	Critical for *M. javanica* parasitism, may manipulate nuclear functions of the host cell	Nuclei of giant cells during nematode parasitism	Lin et al., 2013
*Gr-vap1*	*G. rostochiensis*	Venom allergen-like effector protein trigger a defense-related programmed cell death in tomato plants harboring Cf-2 and Rcr3^pim^	Subventral esophageal gland of preparasitic J2	Lozano-Torres et al., 2012
*Mi-eff1*	*M. incognita*	*M. incognita* EFFECTOR- may manipulate nuclear functions of the host cell	Dorsal oesophageal gland and targeted to the nuclei of the NFSs	Jaouannet et al., 2012
*Hg-MMP*	*H. glycines*	Metalloproteinases, role in hatching in other organisms while in cyst nematodes unknown	Eggs primarily containing fully developed J2	Kovaleva et al., 2004
*map-1*	*M. incognita*	Involved in early steps of recognition between plants and nematodes.	Amphidial secretions of the nematode	Semblat et al., 2001
*Hg30C02*	*H. glycines*	The effector protein interact with a plant β-1,3-endoglucanase to suppress host defense and promote parasitism	Single enlarged dorsal oesophageal gland cell of J3 stage	Hamamouch et al., 2012
*Hs30C02*	*H. schachtii*	Interact with a plant β-1,3-endoglucanase to suppress host defense and promote parasitism	Targeted for secretion outside the nematode gland cell into plant cells	Hamamouch et al., 2012
*MiTPI*	*M. incognita*	Triosephosphateisomerase-unknown	Subventral oesophageal glands	Bellafiore et al., 2008
*Rs-ttl-1 to -4*	*Radopholus similis*	Transthyretin-like genes-unknown	Rs-ttl-1 (the tissues around the vulva), Rs-ttl-2 (the ventral nerve cord)	Jacob et al., 2007
*Mi-gsts-1*	*M. incognita*	Glutathione-stransferases (GSTs)- may be required for correct nematode development after gall formation	Subventral glands of J2 and J3 stages	Dubreuil et al., 2007
*Hs-syv46*	*H. schachtii*	*CLE*-like gene- important for parasitism	Dorsal oesophageal gland cell of parasitic life stages	Patel et al., 2008
*Hg-syv46*	*H. glycines*	Functional similarity of *Hg-syv46* to plant-secreted CLE ligands that may be required for differentiation of NFSs	Dorsal oesophageal gland cell of parasitic life stages	Wang et al., 2005
*Mi-asp2*	*M. incognita*	Encodes an active aspartic protease, a role for the protein in pre-digestion of peptidic nutrients is unlikely since J2s do not feed during migration	Subventral glands of J2 stage	Vieira et al., 2011
*Mi 6D4*	*M. incognita*	Unknown	Both the subventral and dorsal glands of parasitic stages	Davis et al., 1992
*Hg-gp*	*H. glycines*	RNAi silencing of this gene disrupts both normal rates of parasite establishment and sexual fate	Single dorsal gland cell only	Bakhetia et al., 2007
*MhTTL2*	*M. hapla*	Putative role in nematode nervous system during parasitism	Amphids	Gleason et al., 2017
*Mh265*	*M. hapla*	Modulation of plant basal immune responses	Subventral esophageal gland cells	Gleason et al., 2017
*MgGPP*	*M. graminicola*	RNAi silencing resulted in substantially increases the resistance of rice to *M. graminicola*	Subventral esophageal gland cells	Chen et al., 2017

Some effector proteins are involved in molecular mimicry of the host proteins in different ways. Various genes coding for these effectors have been reported to promote nematode parasitism by modulating plant defense pathways. For instance, SPRYSEC effectors (Huang et al., 2006b; Rehman et al., 2009b; Sacco et al., 2009; Postma et al., 2012), CLAVATA3 (CLV3)/ESR (CLE)-like (Wang et al., 2005; Patel et al., 2008; Lu et al., 2009; Wang J. et al., 2010; Wang J. Y. et al., 2010), *Mi-EFF1*, calreticulin *Mi-CRT*, and *Hs19C07* are reported for mediating the compatible plant-nematode interactions (reviewed by Ali et al., 2015).

The nematode effectors are able to target the host proteins directly for compatible plant-nematode interactions by modulating plant defense responses. Chorismate mutases (CM) are regulatory enzymes, involved in host–parasite interaction by mimicking the plant regulatory pathways (Lambert et al., 1999; Doyle and Lambert, 2003; Jones et al., 2003). *M. javanica* chorismate mutase 1 (MjCM-1) predominantly regulates the auxin pathway by lowering the IAA levels in plant cells by triggering a competition for chorismate metabolism. This results in an alteration of chorismate-derived metabolites and ultimately, in plant cell expansion (Doyle and Lambert, 2003). It was postulated that MjCM-1 may be involved in facilitating nematodes to establish a host-parasite relationship with the plant by manipulating auxin pathways. Very recently, a novel *H. schachtii* tyrosinase-like effector protein (Hs-Tyr) has been identified (Habash et al., 2017). Hs-Tyr, localized in the nematode esophageal gland, was found to be important for normal infection and establishment of *H. schachtii* on Arabidopsis roots. Tyrosinases are copper monooxygenases that might be involved in the synthesis of phenolic compounds. Expression of Hs-Tyr in Arabidopsis led to changes in the homeostasis of auxin and the ethylene precursor aminocyclopropane-carboxylic acid (Habash et al., 2017).

**Figure 2:**
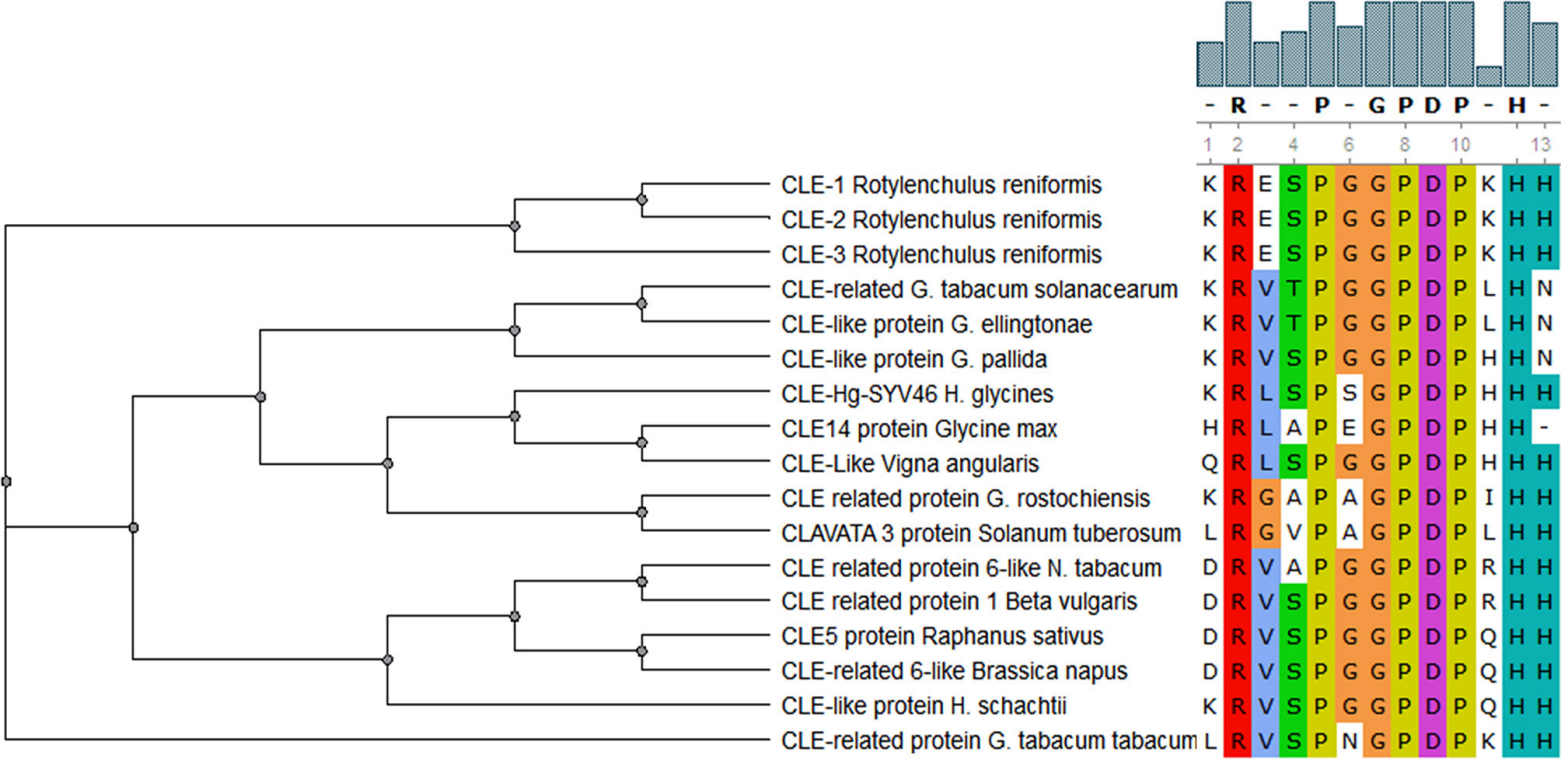
Comparison of CLE motif in CLAVATA/ESR (CLE) peptides from different PPNs and plant species. This demonstrates highly conserved amino acid residues in this motif with six substitutions in total (one, two, or three). It reads xRxxPxGPDPxHx at 95% level of similarity cutoff.

A variety of CLAVATA/ESR (CLE) peptides inhibit or promote cell differentiation in meristems of the plants; however, this family of signaling peptides is not limited to the plant kingdom but also found in nematodes (Mitchum et al., 2008). These peptides contain highly conserved LxLxxxLILxLLLxS and KRLVPSGPNPLHH motifs that are found both in nematodes and plants (Olsen and Skriver, 2003). The CLE motif sequence of CLE protein from *H. glycines, HgSYV46*, is very similar to that of soybean (*G. max*) and *Vigna angularis* (Figure 2). Similarly, CLAVATA/ESR (CLE) peptides from *G. rostochiensis* and potato and *H. schachtii* and sugar beet fall in the same cluster based on amino acid sequence for CLE motif (Figure 2). This significant sequence and functional similarity between plant and cyst nematode CLEs proposes that the nematode CLEs are potential mimics of repressing cell proliferation and promotion of cell differentiation in plants. This is a key feature in the resemblance of syncytium development with that of xylem differentiation (Fisher and Turner, 2007).

The first CLE gene present in the animal kingdom was identified from the parasitic soybean cyst nematode *H. glycines* and named *HgSYV46* (Wang et al., 2005). The encoded protein contains a putative signal sequence at its N terminus and a 14 AA CLE domain near its C terminus and is specifically expressed within the dorsal esophageal gland cell (Wang et al., 2005; Wang and Fiers, 2009). Four out of five potato cyst nematode CLE genes expressed in esophageal gland cells contain multiple CLE motifs which also have been found in rice, *Medicago*, and bread wheat multidomain CLE genes (Oelkers et al., 2008; Lu et al., 2009). A *G. rostochiensis* effector GrCLE1 is processed by host plant proteases and after processing binds directly to the plant CLE receptors CLV2, BAM1, and BAM2 to mimic their function (Guo et al., 2011). Wubben et al. (2015) have described putative CLE genes from *Rotylenchulus reniformis* (Linford and Oliveira) named *Rr-cle-1, Rr-cle-2*, and *Rr-cle-3* similar to those of *Heterodera* CLE genes. They suggested that these CLE genes may be involved in governing host specificity, range, and facilitation of syncytium formation. This further advocates that these CLE proteins are involved in the proliferation and differentiation of plants cells during the development of NFSs. Kiyohara and Sawa (2012) reviewed the molecular properties of CLE peptides from plants and nematodes and their modes of action that may provide further insight into plant cell-cell communication, which could also be applied to manipulate plant-nematode interactions.

The RNAi mediated suppression of transcripts of 16D10 effector peptide from *M. incognita* resulted in impaired development of the nematode showing that this gene is important for successful infection and parasitism (Huang et al., 2006a). Similar results have been shown recently, as dsRNA mediated gene silencing of 16D10 effector from *M. incognita* significantly inhibited the infection in transgenic grape hairy roots (Yang et al., 2013). Although, 16D10 from *M. incognita* is similar to CLE peptides, however, its mode of action is different from the CLE and CLE-like proteins of cyst nematodes. The 16D10 peptide is mostly active in the cytoplasm of infected plant cells compared to uninfected ones via binding to a plant SCARECROW-like transcription factor (Huang et al., 2006b). *In planta* gene silencing using RNAi of the 16D10 effector gene established broad resistance in potato against all *Meloidogyne* species (Dinh et al., 2015). However, suppression of 16D10 transcripts did not interfere with attraction and invasion of *M. incognita* second-stage juveniles toward potato roots. This suggests that 16D10 peptide is important for post invasion success of *M. incognita* on potato roots.

Using the yeast two-hybrid assay, a direct interaction was found between 10A06 effector peptide and a plant spermidine synthase 2 (SPDS2), an enzyme involved in polyamine (spermidine) synthesis in plants (Hewezi et al., 2010). This interaction resulted in an elevated amount of SPDS2 protein in the infected plant cells. This brings about morpho-physiological changes in the cell caused by an altered amount of polyamines. Additionally, the enhanced antioxidant protection and disruption of SA signaling are key features modulated by nematode effector 10A06 (Hewezi et al., 2010). Similarly, the cyst nematode effector protein, 30C02 specifically interacts with Arabidopsis β-1,3-endoglucanase (*At4g16260*) to promote successful parasitism. This was confirmed by *in planta* silencing of 30C02 that led to up-regulation of *At4g16260* which in turn significantly decreased nematode infection on Arabidopsis roots (Hamamouch et al., 2012). Likewise, a newly identified effector protein from *M. incognita* (Mi-EFF1) is targeted to nuclei of the NFSs where it possibly manipulates the nuclear functions of the host cell (Jaouannet et al., 2012). However, the exact molecular function of Mi-EFF1 is still unknown. A similar novel nematode effector protein, MJ-NULG1a that is targeted to giant cell nuclei and plays a critical role in *M. javanica* has been found recently (Lin et al., 2013). But the exact function of this protein remains elusive. Another root-knot nematode calreticulin Mi-CRT, secreted in the apoplasm by the nematode, is demonstrated as a key effector involved in plant basal defense suppression by down-regulation of genes involved in defense responses of the plants (Jaubert et al., 2002, 2005; Jaouannet et al., 2013). The overexpression of Mi-CRT in Arabidopsis has resulted in suppression of the induction of defense related genes and callose deposition after treatment with the PAMP elf18 (Jaouannet et al., 2013). Similarly, a calcium binding calreticulin protein (Bx-crt-1) expressed in the esophageal gland of *B. xylophilus* has been found to be involved in cell-to-cell trafficking and differentiation of nematode feedings cells thus helping the nematodes for the establishment on the plants (Li et al., 2011). Moreover, a calreticulin (*Rs-CRT*) from the migratory nematode *Radopholus similis* [(Cobb, 1893) Thorne, 1949] expressed in esophageal glands and gonads of females and males, the intestines of different juveniles and eggs was found essential for the reproduction and pathogenicity in tomato roots (Li et al., 2015). This demonstrates that silencing of calreticulin genes could be used as potential tool for the development of transgenic plants with enhanced resistance against both sedentary and migratory PPNs.

A nematode secreted ANNEXIN like gene (*Hs4F01*) from *H. schachtii* showing functional similarity to plant annexin has been added to the expanding list of molecular mimics secreted by nematodes (Patel et al., 2010). The annexins bind calcium and phospholipids and are involved in a variety of cellular and physiological processes associated with abiotic stress responses in plants. Hs4F01 annexin-like effector secreted into host root cells may mimic plant annexin function during the parasitic interaction (Patel et al., 2010). A recent study has shown that the cereal cyst nematode *H. avenae* annexin like protein (Ha-annexin) is localized in the nucleus of plant cells and suppresses plant defense (Chen C. et al., 2015). A transgenic wheat line containing a host induced gene silencing construct revealed impaired nematode development. Moreover, transient expression of Ha-annexin resulted in suppression of the host hypersensitive response triggered by BAX protein and various pathogen associated molecular patterns (PAMPs) such as flagellin (Chen C. et al., 2015). It is proposed that this protein binds with the promoters of the defense related genes to suppress the transcription of these genes for infection and parasitic success.

The development of nematode feeding cells is highly associated with auxin accumulation in the NFSs (Grunewald et al., 2009). Sugar beet cyst nematode, *H. schachtii*, effector protein 19C07 has the ability to bind to an auxin transporter LAX3 in Arabidopsis to control NFS development by modulating auxin influx in syncytia (Lee et al., 2011). Auxin and cytokinin trigger the development and proliferation of protophloem around the giant cells and metaphloem around the syncytia (Absmanner et al., 2013). However, MjCM-1 is proposed to lower the IAA level in plant cells by triggering a competition for chorismate that leads to an alteration of chorismate-derived metabolites which, in turn, ultimately results in plant cell expansion allowing nematodes to establish a compatible interaction with the plants (Doyle and Lambert, 2003). Similarly, cytokinins are very important hormones for successful development of NFSs. In a recent study, cytokinin deficient mutants of Arabidopsis resulted in reduced susceptibility against beet cyst nematode (Siddique et al., 2015). It was also observed that *H. schachtii* contains a cytokinin-synthesizing isopentenyltransferase gene which modulates virulence, cell division in plant root and establishment of syncytia in Arabidopsis.

Hyper-Variable Apoplastic (HYP) effector genes belong to a novel gene family which was discovered in *G. pallida*. These are organized in three different subfamilies and the encoded proteins contain subfamily specific tandem repeats at the C-terminus. The genes are expressed in amphidial sheath cells while the proteins were found at the interface between plant and nematode, between the nematode and the syncytial cell wall. HYP effectors were also identified in other cyst nematodes but not in *Meloidogyne* species. How these effectors operate is not yet known but the members of this family (*Gp-hyp*) were found to be important for the development of nematode susceptibility in potato hairy roots (Eves-van den Akker et al., 2014).

Some effector proteins are targeted to the nuclei of the plant cells to reprogram the expression of various plant genes. A *M. incognita* effector protein (7H08) was found to be imported into the nuclei of plant cells after being delivered by the nematode into giant cells. According to the authors, this was the first report of a nematode effector that has transcriptional activation ability in the plant nucleus (Zhang et al., 2015). It depicts that nematode effectors can act as transcription factors to regulate the expression of plant genes.

**Figure 3:**
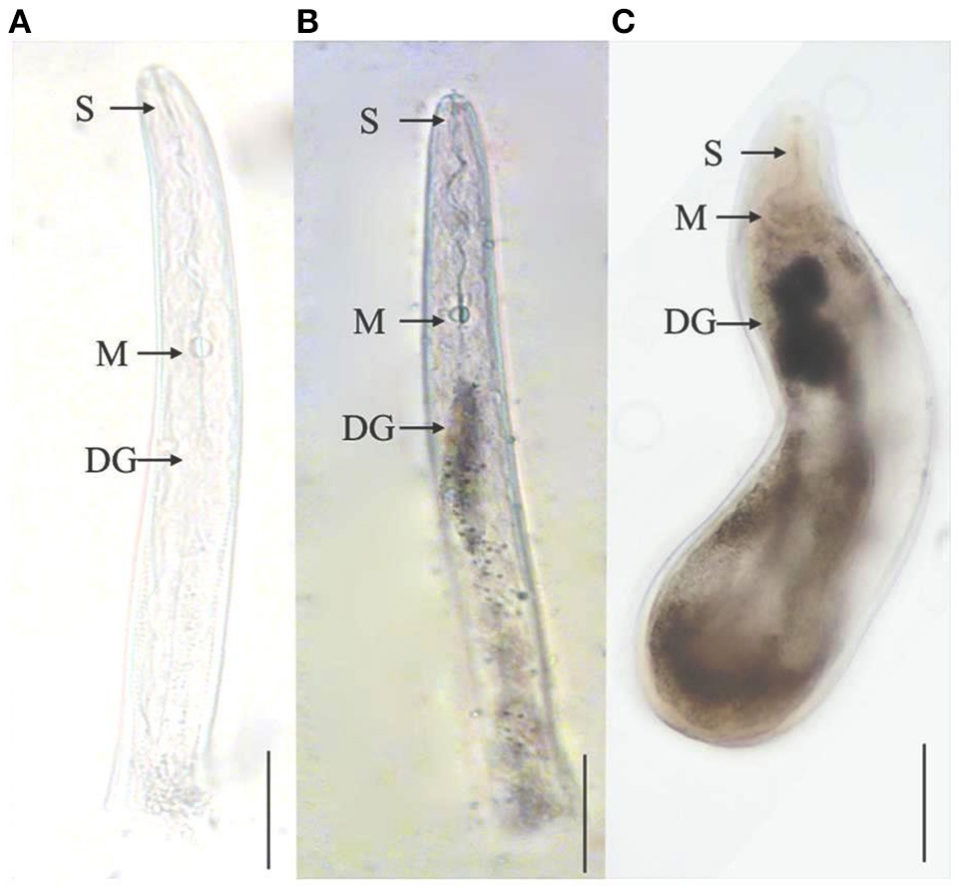
Localization of effector protein Misp12 in different parasitic stages of *M. incognita*. **(A)** The sense Misp12 DIG-labeled cDNA probes as a negative control in parasitic second-stage juveniles. **(B,C)** Misp12 is localized in the dorsal esophageal gland (DG) of parasitic 2nd stage juveniles and females. The DG, metacorpus (M), and stylet (S) are indicated with arrows. Scale bar = 50 μm. The figure is reproduced from Xie et al. (2016) with permission from the authors.

A recent study revealed that *M. graminicola* effector, MgGPP, is exclusively expressed in subventral esophageal gland cells and up-regulated in 2nd stage juveniles (Chen et al., 2017). Based upon RNAi silencing and overexpression studies of MgGPP, it was reported that MgGPP is required for a successful nematode parasitism in rice. N-glycosylation of MgGPP was found to be essential for suppression of the host resistance response. Most of the effector proteins are reported to be active in the early stages of PPNs. However, an effector gene, *Misp12*, has been found in *M. incognita* to be involved in later stages of parasitism on plant roots (Figure 3; Xie et al., 2016). *In planta* virus-induced RNA silencing of this effector gene resulted in a significant decrease of parasitic ability of *M. incognita*. Suppression of *Misp12* resulted in up-regulation of defense related genes of jasmonic acid (JA) and salicylic acid (SA) pathways, while overexpression of *Misp12* promoted down-regulation of genes involved in SA pathway. This suggests that *Misp12* is associated with the suppression of defense response of the plants.

Other nematode effector proteins can disarm resistance proteins deployed by the plants. It has been shown that the pinewood nematode *B. xylophilus* employs a multilayered detoxifying approach by using various effectors in a systematic way for protection against host defense responses during the infection process (Espada et al., 2016).

Similarly, a *M. incognita* effector protein MiMsp40 was found to be important for nematode parasitism on Arabidopsis roots (Niu et al., 2016). The overexpression and silencing of MiMsp40 in Arabidopsis demonstrated nematode success and suppression, respectively, which was coupled with reduction in callose deposition and elf18-triggered immunity. Moreover, its transient expression revealed inhibition in Bax-triggered defense-related programmed cell death and Effector Triggered Immunity (ETI) cognate elicitors R3a/Avr3a. This proposed that this novel effector is very vital for the suppression of both Pattern Triggered Immunity (PTI) and ETI mediated plant defenses to facilitate nematode parasitism (Niu et al., 2016).

## Effectors uncovered by the plant resistance system

If effectors are recognized by the plant resistance system, especially R proteins, they are called avirulence (Avr) proteins and their recognition, either directly or indirectly, leads to ETI. There are several examples of nematode proteins which act as Avr proteins for conferring gene-for-gene resistance to the host plants. Some of these nematode effectors lead to resistance responses in plants. MiCg-1 (Gleason et al., 2008), Hg-cm-1 (Bekal et al., 2003; Lambert et al., 2005; Castagnone-Sereno et al., 2009), MiMAP-1.2 (Castagnone-Sereno et al., 2009), Gp-Rbp-1 (Blanchard et al., 2005; Sacco et al., 2009), Gr-VAP1 (Lozano-Torres et al., 2012), and SPRYSEC-19 (Rehman et al., 2009b; Postma et al., 2012) are some important examples of nematode effectors which are involved in incompatible plant-nematode interactions.

The MiMAP-1.2 protein secreted by *M. incognita* was specifically expressed in lines avirulent on *Mi-1* resistant tomato plants (Semblat et al., 2001; Castagnone-Sereno et al., 2009). A similar behavior was shown by Hg-CM-1 protein against soybean cultivars. However, the corresponding resistance genes for these candidate *Avr* genes and their particular functions are still unknown (Bekal et al., 2003; Lambert et al., 2005). Another gene (*Cg-1*) from *M. incognita* was also described as an *Avr* gene against the *Mi-1* gene in tomato (Gleason et al., 2008). This was confirmed by soaking the nematode juveniles of *M. incognita Mi*-*1*-avirulent strain in dsRNA corresponding to part of the predicted *Cg-1* transcript. This resulted in virulence of these juveniles on tomato carrying the *Mi-1* gene, strongly suggesting that *Cg-1* expression in the nematode is required for *Mi-1*-mediated resistance.

Members of the SPRYSEC family were also found to be involved in incompatible plant-nematode interaction (Rehman et al., 2009b). The *SPRYSEC-19* gene has been isolated from *G. rostochiensis*, the protein of which interacts with the LRR region of a novel CC-NBS-LRR protein (SW5-F) that corresponds to a resistance gene cluster similar to the SW5 family. However, SPRYSEC-19 did not trigger a hypersensitive response in tobacco leaves when it was transiently co-expressed with the SW5-F protein (Postma et al., 2012). In contrast, another SPRYSEC member (Gp-Rbp-1) isolated from *G. pallida* elicits a hypersensitive response when co-expressed with Gpa2, a potato resistance protein, and Ran GTPase Activating Protein 2 (RanGAP2) (Blanchard et al., 2005; Sacco et al., 2009). The variability of potato RanGAP2, on one hand does not affect the recognition of Gpa2; however, on the other hand it enhances the activity of Gpa2 to boost the defense response in potato plants. This suggests that the nematode effector Gp-Rbp-1 is the prime cause of the evolution of RanGAP2 locus in potato as compared to Gpa2 resistance gene (Carpentier et al., 2013). The genome of *G. pallida* contains 299 genes predicted to have one or more SPRY domains, showing a great expansion of this gene family in the white potato cyst nematode as compared to other PPNs (Cotton et al., 2014). The diversity of SPRY domain containing genes in *G. pallida* demonstrates that they might be involved in a wide range of functions, including compatible and incompatible interactions with the host plants. Several *SPRYSEC* genes are differentially expressed between J2s infecting the susceptible cultivar and those infecting the partially resistant lines in potato (Rehman et al., 2009b; Postma et al., 2012).

The venom allergen-like protein (Vap) family is also interesting because of increased transcription of its members during plant infection (Ding et al., 2000; Wang et al., 2007; Kang et al., 2012; Lozano-Torres et al., 2012). An effector protein (venom allergen-like protein Gr-Vap1) was found in *G. rostochiensis* which is reported to interact with the apoplastic cysteine protease Rcr3^pim^ of tomato (Lozano-Torres et al., 2012). In tomato plants containing the resistance gene Cf-2, originally identified as a resistance gene against *Cladosporium fulvum*, this interaction of Gr-Vap1 with Rcr3^pim^ triggers defense-related programmed cell death and resistance to *G. rostochiensis*. VAP proteins from *H. schachtii* when expressed in Arabidopsis induced susceptibility to this nematode but also to unrelated pathogens. It is thought that VAP proteins interact with extracellular papain-like cysteine proteases to suppress programmed cell death mediated by surface-localized immune receptors (Lozano-Torres et al., 2014). Pine wood nematode *B. xylophilus* secreted the potential host mimicry proteins/effectors which closely resemble host pine proteins (Shinya et al., 2013). These proteins might have been acquired by co-evolution of host and parasite and might mimic the host defense systems for compatible and incompatible interactions. A Vap gene, *BxVap-1*, has also been identified and characterized in *B. xylophilus*, which might be involved in suppressing defense mechanism of the pine tree (Kang et al., 2012). In addition to co-evolution of genes in the nematodes and their host plants, convergent evolution of effectors from divergent parasites of plants and animals has also occurred. A recent study indicated that *H. glycines* uses HgGLAND18 effector protein for the suppression of both basal and hypersensitive innate immune responses. This protein contains a unique N-terminal domain very similar to that of malaria causing *Plasmodium* spp. (Noon et al., 2016). Moreover, an effector protein, GrEXPB2 was reported to suppress immunity-associated cell death induced by an extracellular elicitor, necrosis and ethylene-inducing protein-Like Protein (PiNPP) from *Phytophthora infestans* and NB-LRR immune receptor Rx (Ali S. et al., 2015). This revealed that apoplastic and cytoplasmic NB-LRR mediated immune responses conditioned by PiNPP and Rx, respectively, are inhibited by GrEXPB2. Furthermore, plant cell death mediated by NB-LRR Bs2 was also suppressed by GrEXPB2 and its cognate effector AvrBs2 (Bs2/AvrBs2) transiently expressed in the leaves of *N. benthamiana* and *N. tabacum* (Ali S. et al., 2015). However, GrEXPB2 was found to be involved in the elicitation of defense responses in a number of potato and tomato lines. This dual nature of GrEXPB2 function in suppressing and inducing plant resistance is interesting and needs extensive research.

The report from Iberkleid et al. (2013) has provided evidence that Fatty Acid-and Retinol-Binding Protein Mj-FAR-1 aids the process of infection through the suppression of host lipid-based defense mechanisms. They have shown that the tomato roots overexpressing *Mj-FAR-1* led to the down-regulation of JA responsive genes like proteinase inhibitor (Pin2) and γ-thionin, demonstrating the probable role of Mj-FAR-1 in modulating the lipid based signaling *in planta*. Very recently *in planta* silencing of two pioneer genes msp-18 and msp-20 supported lower numbers of *M. incognita* on transgenic egg plants (Shivakumara et al., 2017). Moreover, suppression of these pioneer genes resulted in the down-regulation of cell wall modifying enzymes (CWME), i.e., Mi-pg-1 and Mi-pel, in females developing in the best transgenic events as compared to the control. This suggests that transcriptional repression of CWME genes due to silencing of msp-18 and msp-20 could protect the plants against the root knot nematode. Mantelin et al. (2015) provided the insights into the involvement of effectors in activation and suppression of host innate immune responses. However, mostly the effectors are reported to target host defense and nuclear functions to establish NFSs in plant roots.

## Nematode effectors recruit plant genes for a compatible interaction

PPNs use a variety of effectors to induce NFSs and to maintain the function of these feeding sites. We have already discussed different effectors that are involved in these processes. However, during the recent years, many plant genes have been identified as being manipulated by the nematodes for the above purposes while it is still unknown which effectors might be involved in the expressional modulation of these genes.

In addition to injecting their own CWDEs and related proteins, nematodes also activate the CWDEs of plants, such as endo-1,4-β-glucanases, cellulases, pectate lyases, expansins, and tubulins which facilitate the nematodes to modify and degrade plant cell walls to support the nematode invasion and ultimate establishment of NFSs (Goellner et al., 2001; Wieczorek et al., 2006, 2008, 2014; Szakasits et al., 2009; Barcala et al., 2010; Banora et al., 2011). The knockout mutants of two endo-1,4-β-glucanases, which were highly up-regulated in syncytia, revealed less susceptibility of Arabidopsis in response to the beet cyst nematode (Wieczorek et al., 2008). This reveals that in addition to their own pectate lyases, nematodes require plant pectate lyases to develop NFSs. The expression of expansin family genes was also reported to be activated in syncytia induced by the beet cyst nematode in Arabidopsis (Wieczorek et al., 2006). Similarly, the expression of pectate lyase like genes *PLL18* (*At3g27400*) and *PLL19* (*At4g24780*) was found to be induced by *H. schachtii* and *M. incognita* in syncytia and giant cells, respectively (Wieczorek et al., 2014). Knock out T-DNA insertion mutants of both of these genes demonstrated their significant role in the development of syncytia but not in case of giant cells. Additional plant genes that are strongly up-regulated in nematode NFSs include an ATPase gene from Arabidopsis, *At1g64110*, which was highly upregulated in syncytia induced by *H. schachtii* on Arabidopsis roots (Ali et al., 2013b). The knocking down of this gene in syncytia supported lower numbers of nematodes. The *At1g64110* belongs to the meiotic clade of AAA proteins which also include Vps4, katanin, spastin, and MSP1 (Ammelburg et al., 2006). These AAA proteins are known to be involved in remodeling of membranes and shaping of their protein content which are very important components in the development of NFSs (Ali et al., 2013b).

Establishment of NFSs requires hyper-metabolic conditions for morpho-physiological changes occurring in the initial syncytial cells or young giant cells in the early time points of nematode infections. This requires a lot of energy by the plants to execute these processes needing more and more reservoirs of amino acids which are translocated by amino acid transporters. The beet cyst nematode, *H. schachtii*, was able to induce the expression of these transporters in syncytia. It was reported that amino acid transporters are important for the development of NFSs in Arabidopsis (Elashry et al., 2013). Moreover, the WRKY23 transcription factor was up-regulated and involved in the establishment of syncytia induced by *H. schachtii* in Arabidopsis roots (Grunewald et al., 2008). Recently, auxin response transcription factors have been found to be up-regulated in the syncytia induced by *H. schachtii* suggesting their role in determining the maximum size along with maintaining the functional phenotype of mature syncytia; however, their proper function is still to be investigated (Hewezi et al., 2014). Similarly, a study demonstrated that *M. incognita* infection resulted in the up-regulation of Arabidopsis F-box/Kelch-Repeat coding gene *At2g44130* in giant cells (Curtis et al., 2013). This gene was found to be important for nematode development on plant roots as its knock out mutant supported a lower number of nematodes in addition to a more susceptible line overexpressing At2g44130 protein. F-box proteins are crucial for the precise deactivation of cellular regulatory proteins through the process of ubiquitination. This suggested that PPNs induce host F-box proteins to ubiquitinate and degrade plant regulatory proteins, i.e., transcription factors, involved in the process of resistance.

**Figure 4:**
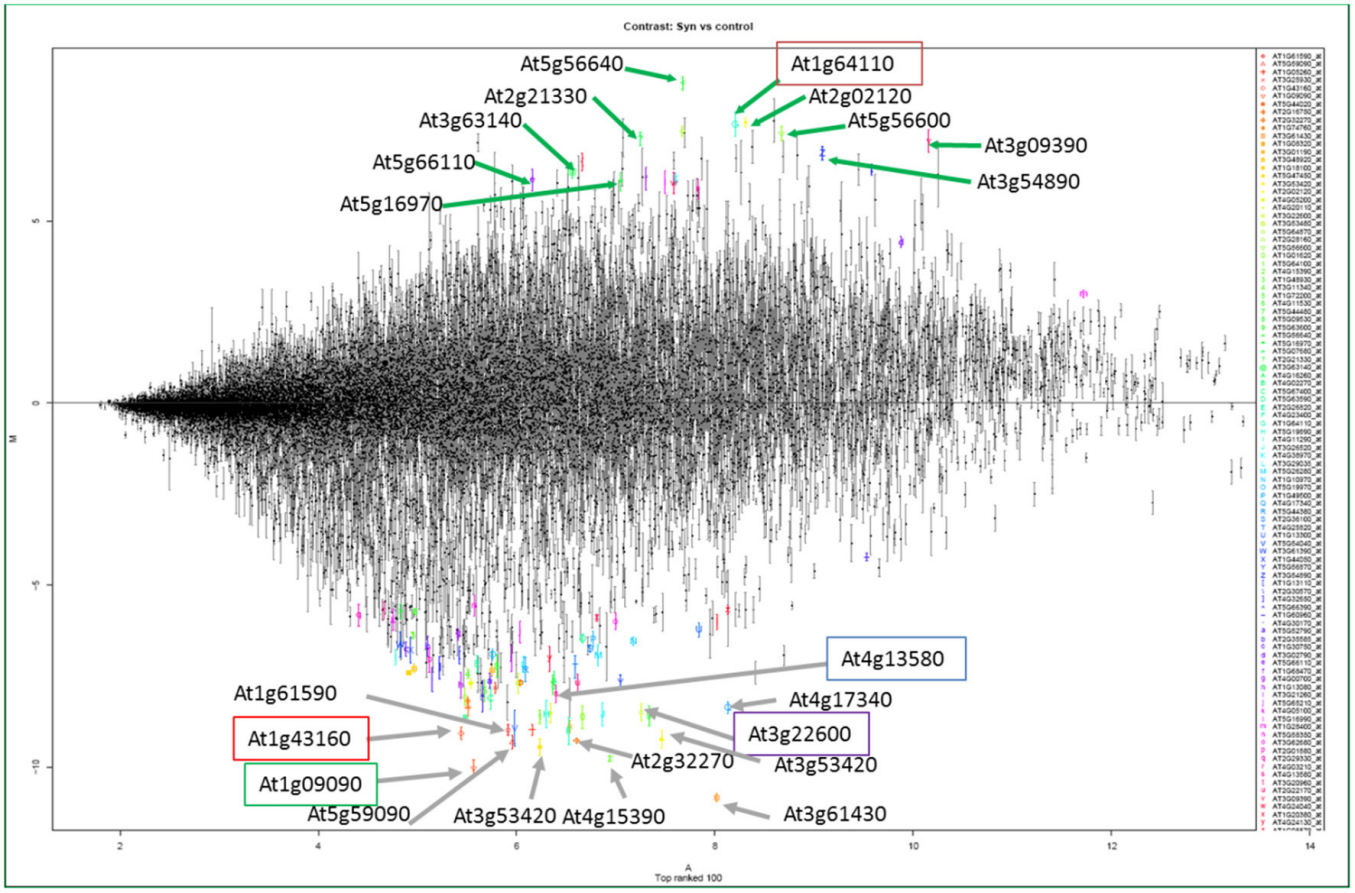
The structure of the transcriptome of syncytia from Szakasits et al. (2009). Strongly up-regulated (above the line) and down-regulated (below the line) genes shown by different colors and letters in the transcriptome of syncytia induced by *H. schachtii* in Arabidopsis roots. The highlighted genes have been characterized in response to *H. schachtii* infection in Arabidopsis (Ali, 2012; Ali et al., 2013a,b, 2014).

Transcriptomes of NFSs compared with control roots confirmed the expression of plant genes related to high metabolic activity in the NFSs (Gheysen and Fenoll, 2002; Puthoff et al., 2003; Bar-Or et al., 2005; Hammes et al., 2005; Jammes et al., 2005; Szakasits et al., 2009). Genes coding for starch synthases, myo-inositol phosphate oxygenase, sucrose transporters, myo-inositol oxygenases, and UDP-glucose dehydrogenase (UGD) related to high metabolic activity are preferentially up-regulated in NFSs during compatible interactions (Hofmann and Grundler, 2007; Hofmann et al., 2007, 2008, 2009a,b, 2010; Afzal et al., 2009; Siddique et al., 2009, 2012; Szakasits et al., 2009; Barcala et al., 2010). An overview of the transcriptome of syncytia induced by *H. schachtii* indicates that *At5g56640*/myo-inositol oxygenase 5 (*MIOX5*) was the top up-regulated gene followed by AAA+ ATPase gene (*At1g64110*), plant defensin gene *Pdf2.1* (*At2g02120*), profilin 3 (*At5g56600*), metallothionein 2A (*At3g09390*), Chlorophyll a-b binding protein 6 (*At3g54890*), fructose-bisphosphatealdolase 2 FBA2 (*At2g21330*), Chloroplast stem-loop binding protein (*At3g63140*), and Heavy metal transport/detoxification superfamily protein (*At5g66110*) (Figure 4; Szakasits et al., 2009). This general overview indicates that most of the genes were involved in the various metabolic pathways of the plants thus rendering hyper-metabolism in syncytia which is quite obvious from the studies of *MIOX5* and AAA+ ATPase genes (Siddique et al., 2009; Ali et al., 2013b).

## PPNs suppress host genes involved in various defense pathways

In addition to recruiting plant genes for compatible interactions, PPNs are smart enough to shut down the defense mechanisms of the plants for establishment of NFSs. Several transcriptome studies of NFSs induced by different PPNs demonstrate the suppression of most of the genes involved in different defense pathways of the plants (Szakasits et al., 2009; Barcala et al., 2010; Kyndt et al., 2012; Ali M. A. et al., 2015). Peroxidases are very important mediators of resistance responses in plants. The transcriptome of syncytia induced by *H. schachtii* demonstrated that out of top hundred most suppressed genes, 14 were coding for peroxidases (Szakasits et al., 2009). This transcriptome revealed the top 10 genes which were down-regulated in 5 and 15 days old syncytia. These genes were *At3g61430* (plasma membrane intrinsic protein 1A, PIP1A), *At1g09090* (RBoHB), *At4g15390* (HXXXD-type acyl-transferase family protein), *At3g53420* (plasma membrane intrinsic protein subfamily PIP2), *At1g43160* (member of ERF/AP2 transcription factor family, RAP2.6), *At3g22600* (Lipid transfer protein), *At2g32270* (zinc transporter, ZIP3), *At5g59090* (subtilase 4.12, SBT4.12), *At1g61590* (putative receptor-like cytoplasmic kinase), and *At3g13580* (disease resistance responsive gene). Most of them are associated with defense related pathways of the plants (Figure 4; Szakasits et al., 2009).

Ethylene response factors are also crucial for transcription regulation of defense related genes (Licausi et al., 2013). Ethylene transcription factor, AtRAP2.6, was one of the most suppressed genes in the syncytia induced by *H. schachtii* in Arabidopsis. The constitutive expression of this gene led to resistance against the beet cyst nematode (Ali et al., 2013a). Moreover, this decrease in susceptibility was coupled with increased expression of JA marker genes and callose deposition. Similarly, WRKY transcription factors are very important modulators of resistance response of plants against biotic and abiotic stresses. WRKY33 is the most important player in the activation of camalexin synthesis pathway in plants against the invading pathogens. It was the most down-regulated gene among the WRKY transcription factors. Syncytia specific overexpression of WRKY33 and constitutive expression of MKK4 led to incompatible plant nematode interaction in the Arabidopsis-beet cyst nematode model system (Ali et al., 2014). However, knockout mutants of WRKY33 and PAD3 resulted in enhanced susceptibility as compared to control which demonstrated that nematodes shut down the phytoalexin pathways for successful parasitism. In addition to WRKY33, other defense responsive WRKYs such as WRKY6, WRKY11, WRKY17, and WRKY25 were also down-regulated in syncytia, which suggests that nematodes have specialized strategies to suppress plant transcription factors that are involved in resistance responses (Ali et al., 2014).

## PPNs manipulate RNA silencing pathways for successful parasitism

RNA silencing pathways are very important modulators of plant growth, development, and responses to biotic and abiotic stresses. A variety of RNA silencing pathways are involved in plant defense responses against invading pathogens such as viruses and bacteria. Walsh et al. (2017) recently demonstrated the importance of these pathways in plant nematode interactions. When viral RNA suppressors were expressed in tobacco plants, they became more susceptible to *M. incognita*. MicroRNAs (miRNAs) are one of the key players involved in RNA silencing pathways of the plants. These miRNAs are involved in the regulation of a variety of plant processes ranging from seed development to plant responses against biotic and abiotic stresses (Sunkar, 2012). It has been reported that the Arabidopsis miRNA396 interacts with GRF1/GRF3 transcriptios factors to regulate the reprogramming of root cells during beet cyst nematode infection (Hewezi et al., 2012).

Twenty miRNAs were found to have diverse expression patterns between susceptible and resistant soybean lines during the interaction with *H. glycines* (Li et al., 2012). Similarly, a recent study confirmed that the majority of miRNAs from tomato roots infected with *M. incognita* were significantly up-regulated during a susceptible interaction (Kaur et al., 2017). Moreover, a negative correlation was observed in the up-regulated miRNAs (miR156, miR159, miR164, and miR396) and their target transcription factors, SBP, GAMYB-like, NAC, and GRF1, respectively. It suggests that miRNAs play a vital role in the regulation of transcription factors involved in plant development and resistance to favor compatible plant-nematode interactions at molecular level.

## The nematodes influence ROS pathways for compatible interaction

Reactive Oxygen Species (ROS) are a byproduct of metabolism and can be destructive for the cells. Therefore, these reactive molecules have to be detoxified by various enzymes. However, ROS are also signaling molecules. Plants produce ROS to activate their defense responses against the pathogens and to stimulate programmed cell death (PCD) or the hypersensitive response (HR) to detain and kill the invading pathogens at the site of infection. The production of ROS is highly dependent on the concentration of different hormones, especially SA, which is the main stimulator of HR at the site of pathogen invasion and surrounding cells (Durner et al., 1997; Durrant and Dong, 2004). Production of ROS is one of the earliest responses of the plants to infections (Torres, 2006). Kandoth et al. (2011) showed up-regulation of various genes involved in ROS biosynthesis in *H. glycines* resistant lines harboring the *Rhg1* gene which confers resistance against soybean cyst nematodes. Similarly, Beneventi et al. (2013) suggested that in a resistant line of soybean, auxin-induced ROS production is positively correlated with Ca^2+^ conductance across the cell membranes, possibly with the participation of plant annexins in the terminal growth points of the roots. Furthermore, DELLA-like proteins were found to be involved in auxin regulated ROS signaling, which controls resistance responses of resistant soybean plants against parasitic nematodes (Beneventi et al., 2013).

Nematodes have specialized enzymes called superoxide dismutases (SODs) in their secretions to detoxify and minimize the effects of ROS. Several genes have been discovered, which code for SODs in root knot nematodes (Bellafiore et al., 2008; Roze et al., 2008). Nematodes also scavenge ROS by using enzymes such as ascorbate peroxidases, cytochrome C-peroxidases, catalases, thioredoxins, and glutathione peroxidases, which protect cells from ROS-catalyzed damage (Campos et al., 2005; Bellafiore et al., 2008).

Most of the times, synthesis of ROS is regulated by SA accumulation in the cell which leads to PCD in response to pathogen infection in plants (Draper, 1997; Overmyer et al., 2003). However, ROS production through SA pathway antagonizes with that of the Respiratory Burst Oxidase Homologs (RBOH) dependent pathway in Arabidopsis (Torres et al., 2005). A recent study demonstrated that nematodes are able to manipulate the plant ROS pathway to develop a compatible interaction with the plant roots (Siddique et al., 2014). In this study, it was reported that *H. schachtii* stimulates the Arabidopsis RBOH genes *RBohF* and *RBohD* to produce ROS that in contrast to defense induction results in the inhibition of PCD to promote establishment of the nematodes on plant roots. Similarly, the cloning, expression and functional analysis of a peroxidase gene belonging to the peroxiredoxin family from *G. rostochiensis* revealed that the peroxiredoxins could play an important role in protection of the parasite from plant defense responses by a series of redox reactions (Robertson et al., 2000).

## Effectors mediate post-translational modifications for compatible interactions

In addition to various other strategies, PPNs modulate post-translational modifications (PTMs) in plants by interacting with plant proteins involved in various PTM pathways. These modifications include phosphorylation (Hewezi et al., 2015), ubiquitination (Chronis et al., 2013), glycosylation (Chen S. et al., 2015), proteolysis (Lozano-Torres et al., 2014; Noon et al., 2016), and histone modifications (Jones et al., 2009). Phosphorylation and dephosphorylation are very important PTMs and are the main players of signaling cascades in living organisms. Hewezi et al. (2015) reported the involvement of an effector protein (10A07) in targeting the plant transcriptional machinery for its promotion of parasitism. They further demonstrated that the 10A07 protein physically interacts with Interacting Plant Kinase (IPK) and the IAA16 transcription factor. This results in phosphorylation of the 10A07 protein at serine number 144 and 231 to mediate its trafficking from the cytoplasm to the nucleus (Hewezi et al., 2015). It has been shown that CLV2 and CORYNE (CRN), members of the receptor kinase family, are required for nematode CLE signaling and are important for both the successful nematode infection and the formation and maintenance of nematode-induced syncytia (Replogle et al., 2011). Recently, it was demonstrated that two additional receptors, CLV1 and Receptor-Like Protein Kinase 2/TOADSTOOL2 (RPK2), are able to transduce the CLV3 signal independent of CLV2/CRN for the maintenance of shoot apical meristem in Arabidopsis (Replogle et al., 2013). Both of these receptors also play an important role in nematode CLE perception.

Ubiquitination involves the attachment of ubiquitin to a substrate protein in eukaryotic cells. The ubiquitin extension protein was found to be only prevalent in cyst nematodes (Tytgat et al., 2004). In total, 12 Hs-Ubi1 homologous were found in cysts of *H. schachtii* but none from root-knot nematodes. It indicated that the short C-terminal polypeptide of Hs-UBI1 could be important for the development of the syncytium but not in giant cell formation (Tytgat et al., 2004). Likewise, a ubiquitin carboxyl extension protein, GrUBCEP12 secreted by the potato cyst nematode *G. rostochiensis* was reported to be processed into free ubiquitin and a CEP12 peptide to develop successful parasitism (Chronis et al., 2013). RNAi mediated silencing of GrUBCEP12 in plants displayed reduction of susceptibility in potato plants against *G. rostochiensis*. This revealed that ubiquitination is an important PTM which promotes compatible plant-nematode interaction.

Glycosylation is a complex PTM that involves the addition of carbohydrate molecules to a protein through covalent bonding. This modification significantly affects the biophysical properties of proteins. A multidomain effector protein from *G. rostochiensis* (GrCLE1) was processed into 12-amino acid arabinosylated glycopeptides through the process of glycosylation (Chen S. et al., 2015). This glycosylation lead to enhanced resistance of this peptide against hydrolytic degradation and high affinity binding to potato CLV2-like receptor (StCLV2) as compared to its non-glycosylated counterpart. This suggested the importance of these PTMs for the promotion of compatible plant nematode interactions. Hewezi (2015) has reviewed the importance and putative role of PTMs like phosphorylation, ubiquitination, glycosylation, proteolysis, and histone modifications in supporting nematode parasitism in plants.

## Conclusions and outlook

As a consequence of enormous yield losses in crop plants imposed by the PPNs, the understanding of plant nematode interactions is becoming of utmost importance. PPNs use multiple strategies to develop successful parasitism in plants. It has now become clear that nematodes, just like other plant pathogens, produce a range of different effectors and suppressors. Omics approaches are being used to characterize the parasitome of different plant pathogenic nematodes at the genomic level (e.g., Eves-van den Akker et al., 2014; Eves-Van Den Akker et al., 2016), the transcriptome level using RNAseq (e.g., Paiva et al., 2013), and the proteome level (e.g., Shinya et al., 2013). Various bioinformatical approaches such as OrthoMCL (Chen et al., 2006) could be very important for computational identification of putative effector proteins (Rutter et al., 2014).

Now that a large number of putative effectors and suppressors are being identified, specific emphasis has to be put on the identification of their plant targets. Much of that work is being done in the model plant Arabidopsis using all the resources available for this species. In other plant species, virus-induced gene silencing (VIGS) could provide a tool to recognize the important genes required for either pathogenic or symbiotic plant-microbe interactions in plants (Kandoth et al., 2013). Genome editing technologies, especially CRISPR-Cas, could also be used for studying potential functions of effector proteins in plant species. However, results obtained with Arabidopsis may not be easily translated to monocots which include the most important crop plants for human nutrition. To study plant-nematode interaction with monocots at the molecular level it will therefore also be important to develop a monocot system such as the rice-*Meloidogyne* model system (Nguyen et al., 2014).

The interaction between plant pathogenic nematodes and their host plants and especially the induction of specific feeding sites is interesting from a biological point of view. But such knowledge will also have practical applications. On one hand, overexpression or downregulation of plant genes that are downregulated or upregulated in feeding sites could lead to enhanced resistance against nematodes (Klink and Matthews, 2009; Ali, 2012; Ali et al., 2017). On the other hand, knowing the function of nematode effectors might also open a way to counteract these. For these transgenic approaches specific promoters would be needed instead of the widely used CaMV-35S promoter (Ali and Abbas, 2016).

## Author contributions

MA conceived, designed, and mainly developed the article; FA contributed in the write up of main body of the review article while HL helped in finalizing the contents and structure of the manuscript, HB reviewed and finalized the article.

### Conflict of interest statement

The authors declare that the research was conducted in the absence of any commercial or financial relationships that could be construed as a potential conflict of interest.
